# Chitosan-Nanoconjugated Hormone Nanoparticles for Sustained Surge of Gonadotropins and Enhanced Reproductive Output in Female Fish

**DOI:** 10.1371/journal.pone.0057094

**Published:** 2013-02-27

**Authors:** Mohd Ashraf Rather, Rupam Sharma, Subodh Gupta, S. Ferosekhan, V. L. Ramya, Sanjay B. Jadhao

**Affiliations:** Central Institute of Fisheries Education, Versova, Mumbai, India; Universidad de Castilla-La Mancha, Spain

## Abstract

A controlled release delivery system helps to overcome the problem of short life of the leutinizing hormone releasing hormone (LHRH) in blood and avoids use of multiple injections to enhance reproductive efficacy. Chitosan- and chitosan-gold nanoconjugates of salmon LHRH of desired size, dispersity and zeta potential were synthesized and evaluated at half the dose rate against full dose of bare LHRH for their reproductive efficacy in the female fish, *Cyprinus carpio.* Whereas injections of both the nanoconjugates induced controlled and sustained surge of the hormones with peak (P<0.01) at 24 hrs, surge due to bare LHRH reached its peak at 7 hrs and either remained at plateau or sharply declined thereafter. While the percentage of relative total eggs produced by fish were 130 and 67 per cent higher, that of fertilised eggs were 171 and 88 per cent higher on chitosan- and chitosan-gold nanoconjugates than bare LHRH. Chitosan nanoconjugates had a 13 per cent higher and chitosan gold preparation had a 9 per cent higher fertilization rate than bare LHRH. Histology of the ovaries also attested the pronounced effect of nanoparticles on reproductive output. This is the first report on use of chitosan-conjugated nanodelivery of gonadotropic hormone in fish.

## Introduction

Natural spawning grounds are undergoing extreme changes as a result of anthropogenic activities, pollution and climate change, leading to decline in fish populations. Moreover, inadequate knowledge of the reproductive and breeding process of several fish species is one of the reasons for inability to overcome depleted fish stock and biodiversity loss. It is a hindrance to realize the full potential of intensification of aquaculture worldwide. Aquaculture production of the world will be 65–85 million tons by 2020 and 79–110 million tons by 2030 as compared to 55 million tons in 2009 [1]. Thus lion’s share of this production will be contributed by fish farming in captive condition. But many cultivable fishes do not reproduce spontaneously in captivity. This leads to inadequate supply of good quality seeds for intensification of aquaculture. Aquaculture production depends on captive broodstock in which full control of biological processes can be exercised through environmental, hormonal and genetic manipulations. In captive condition fishes may exhibit different reproductive responses due to abiotic factors prevailing in the cultured system, which results in stress-induced immune and endocrine disequilibrium. In all fishes reproductive failure such as incomplete vitellogenesis is mostly observed in females, and hence final oocyte maturation and ovulation do not occur [2]. Sometimes, in spite of having oocyte maturation and ovulation, voluntary spawning does not take place. This is due to the complex mating behaviour required for successful spawning and the absence of the natural environmental conditions such as substrate, hydrology and temperature [3]. Thus, there is a need to develop methods for controlling reproductive processes in fishes reared in captivity so that problems related to synchronization; egg collection and seasonal reproduction can be overcome. Nanobiotechnology has enormous potential to revolutionize the field of reproductive biology and overcome barriers to successful reproduction in a number of species, especially via the use of controlled hormonal nanodelivery systems.

The reproductive process can be regulated through controlled release of hypothalmo-pituitary hormones or gonadotropins (GtH) such as follicle stimulating hormone (FSH) and luteinizing hormone (LH). The release of these hormones is controlled by gonadotropin releasing hormone (GnRH). Ovulation and spawning induction therapies have been developed employing pituitary homogenates, purified gonadotropic preparations and more recently, synthetic agonists of the gonadotropin-releasing hormone or other hormones [4]–[6]. All these therapies are expected to enhance surge in GtH. However, sufficient ovulation and spawning does not occur on account of either short lifetime of the GnRH in blood caused by rapid degradation by both specific endopeptidases and non-specific exopeptidases present in pituitary, kidney and liver [7], or insufficient effect of these therapies (effective only in 20–30 percent of females). One way to conquer this limitation and to attain long lasting surge in GtH in the blood is to use multiple injections of GnRH or analogues, but again, multiple injections can place stress on fish. Another approach which has been successfully employed to enhance long lasting surge of GtH in blood is to use a sustained release delivery system [2] through the implantation method. The nanoconjugated delivery of peptides enhances half life of the biomolecule leading to controlled and sustained delivery, as nano-carriers make them able to pass through biological barriers. The coating of biomolecules on nanoparticles of inert metals like gold and silver or encapsulation of nanoparticles of biopolymers provide protection from rapid degradation, targeted delivery and control of the release of bioactive agents.

Chitosan, being cheaper, is one among several compounds which has received a great deal of attention for the preparation of micro and nano-particles for parenteral, nasal, ophthalmic, transdermal and implantable delivery of drugs, proteins, peptides, and gene materials [8]. Chitosan [α (1→4) 2-amino 2-deoxy β-D-glucan] is a cationic polysaccharide obtained from the deacetylation of chitin. It has unique properties such as biocompatibility, biodegradability, low-immunogenicity and non-toxicity [9]. Chitosan nanoparticles are shown to be an attractive alternative to liposomes for the delivery of peptides, proteins, antigen oligonucleotides and genes, since it has the advantages of longer shelf life and generally a higher drug carrying capacity [10] as chitosan exhibits pH dependent solubility [11].

Gold nanoparticles provide non-toxic carriers for drug and gene delivery applications. With these systems, the gold core imparts stability to the assembly, while the monolayer of active compound (such as hormone in this case) allows tuning of surface properties such as charge and hydrophobicity. Nanoparticles of gold are excellent candidates for making nanoconjugates of biomolecules [12]. Many researchers have shown that biologically active substances with amine function can bind strongly with gold nanoparticles [13]. They are biocompatible, bind readily to a large range of biomolecules such as amino acids, proteins, enzymes and DNA, and expose large surface areas for immobilization of such biomolecules [14]. The ability to modulate the surface chemistry of gold nanoparticles by binding suitable ligand has important applications in many areas including drug delivery. Recently, a rapid and mild method for synthesizing magnetite-gold nanoparticles using chitosan was investigated [15].

Gonadotropin-releasing hormone (GnRH), also known as luteinizing-hormone-releasing hormone (LHRH), is a tropic peptide hormone responsible for the release of follicle stimulating hormone (FSH) and LH from the anterior pituitary. GnRH is degraded by proteolysis within a few minutes. The GnRH and its analogues [16] and its controlled release formulations [2] have been extensively used for manipulating reproduction of fishes. The estradiol loaded chitosan nanoparticle delivery has been shown to be more effective in the treatment of various ailments such as estrogen deficient hyperlipidemic condition [17] and long term oestrogen replacement therapy and Alzheimer's disease [18] in rats. However, to the best of our knowledge to date, use of chitosan for fabrication of reproductive hormone nanoparticles has not been reported. The aim of the present study was to prepare nanoconjugates of salmon LHRH with chitosan and chitosan-gold for assessing their efficacy in sustaining serum hormone levels including functional response (ovulation, total eggs and fertile eggs) in test animals. The test species chosen for the purpose was *Cyprinus carpio* (common carp) for its ease of breeding, high fecundity and short breeding season.

## Materials and Methods

### Chemicals

Chitosan from shrimp shell (degree of deacetylation >85 percent, MW 200kDa), salmon luteinizing hormone releasing hormone (amino acid sequence: Glp-His-Trp-Ser-Tyr-Gly-Trp-Leu-Pro-Gly-NH_2_)_,_ gold powder and pentasodium tripolyphosphate were purchased from Sigma-Aldrich Corporation (St. Louis, MN)**.** Commercial enzyme immunoassay (EIA) kits were purchased from Omega Diagnostics Ltd, Scotland, U.K. All chemicals were of reagent grade.

### Preparation of Chitosan Nanoparticles

Chitosan nanoparticles were prepared based on the ionic gelation method [19] of chitosan and tripolyphosphate (TPP) anion with little modification. Briefly, 2 mg of chitosan were dissolved in 100 ml aqueous acidic solution to obtain the cation of chitosan. This aqueous solution was prepared with 80 ml of water, 15 ml of TPP and 5 ml of acetic acid. The solution was subjected to the constant magnetic stirring for 10 min. The pH of the solution was adjusted to 6.5. During the process involving chemical reaction, chitosan undergoes ionic gelation and precipitates to form spherical particles.

### Synthesis of Chitosan- Conjugated Gold Nanoparticles

Chitosan-conjugated gold nanoparticles were synthesized according to the Turkevich [20] procedure of reduction of chloroauric acid with slight modifications. Various chitosan concentrations (0.01%, 0.1% and 0.2%) were used to determine the effect of chitosan concentration on the formation of gold nanoparticles. In each of these solutions 50 µl of chloroauric acid (HAuCl_4_) at a concentration of 1 mg/ml was added. The whole solution was heated at 60–80°C under constant magnetic stirring for 20 min to yield a ruby-red solution. The absorbance of the solution was measured at 520 nm.

### Nano-conjugation of the Salmon Luteinizing Hormone Releasing Hormone

For conjugation of the nanoparticles, a high pressure homogenization process was used. A stock solution of salmon’s LHRH was prepared with 1 mg/1 ml of water. From this stock solution, three different volumes of 50µl, 100 µl and 200 µl were added to chitosan and chitosan-gold nanoparticle solutions. Then concentration of the protein, i.e., LHRH in the solution was measured at 660 nm [21]. After that the solution was homogenized at 35,000 rpm for 10 min and kept for overnight in refrigerator at 4°C. The next day, the solution was centrifuged again at 2000 rpm. Supernatant was collected and the concentration of protein was again determined [21]. The entrapment efficiency (EE) of the hormone in chitosan-LHRH and chitosan-gold-LHRH was calculated using the formula
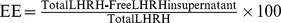



### Characterization of Chitosan and Chitosan-gold LHRH Nanoparticles

The particle size and zeta potential of the nanoparticles were measured using Beckman Coulter Delsa Nano C- NanoParticle Size Analyzer (Brea, CA). The instrument which uses photon correlation spectroscopy gives the particle size in the range of 0.6 nm to 7 µm. A drop of the nanoconjugate suspension sample (about 5µl) was diluted with 5 ml of deionized water. The cuvette was well-shaken by hand and placed immediately inside the sample holder of the particle size analyzer. Once the required intensity was reached, analysis was performed to obtain the mean particle size and polydispersity index (PDI) of the sample. Measurement of the zeta potential gives an idea about the stability of any colloidal system and it was determined based on an electrophoretic light scattering (ELS) technique. The surface morphology (roundness, smoothness and formation of aggregates) was studied by transmission electron microscope (TEM).

### Ethics Statement

The research undertaken complies with the current animal welfare laws in India. The care and treatment of animals used in this study were in accordance with the guidelines of the CPCSEA [(Committee for the Purpose of Control and Supervision of Experiments on Animals), Ministry of Environment & Forests (Animal Welfare Division), Govt of India] on care and use of animals in scientific research. The study was approved by the Board of Studies and authorities of the Central Institute of Fisheries Education (Deemed University), Mumbai-61. As the experimental fish *Cyprinus carpio* is not an endangered fish, the provisions of the Govt of India’s Wildlife Protection Act of 1972 are not applicable for experiments on this fish.

### Animal Procurement, Rearing and Experimental Conditions

The animals (*Cyprinus carpio*) were procured from Aquaculture Farm, Pen, Raigad Dist, Maharashtra (India) and were stocked in a circular tank (1000 L) after giving a prophylactic dip treatment in KMnO_4_ solution (50 mg/L) for 2 min. They were maintained for two months in a backyard hatchery of the Central Institute of Fisheries Education, Mumbai, India prior to the experiment and were fed twice a day with a diet containing 35% crude protein. Water in the tanks was aerated round the clock. One-half of the water was exchanged every week. Water quality parameters in the tank were recorded during the study [22].

### Animal Work and Experimental Treatments

Sexually mature male (average body weight 550g) and female (average body weight 900g) fishes were used for the study. The animal experiment in this study included four treatments with three replicates. Each replicate contained two matured male and two female brood fish in each tank. The first group was maintained as a control in which fishes were not given any injection of hormone. In second group, female broods were given intramuscular injection of salmon LHRH @ 0.2 ml/kg body weight of fish. It was expected that the efficacy of conjugated LHRH nanoparticles would surpass the bare LHRH, hence a solution of chitosan- and chitosan-gold conjugated LHRH was injected at 0.1 ml/kg of fish (half the dose of the bare hormone) in the third and fourth groups, respectively. Solution of each injectable preparation contained 20 µg LHRH and 10 mg domperidone per ml. The injections were given using a 1.0 ml syringe fitted with 22 gauge needle. Blood was collected at the 0^th^, 3^rd^, 7^th^ and 24^th^ hour post-injection in all the groups. However, to avoid stress during blood collection, blood was collected from a single fish on 0^th^ hr and 7^th^ hr while another fish was used for 3^rd^ and 24^th^ hr in each replicate. Post-injection period of 24 hrs was selected for the studies because successful ovulation in this fish [23] and also in majority of tropical fishes can be accomplished within 24 hrs by administration of a single dose of any hormonal preparation including but not limited to LHRH [24]. Various hormones were analysed by enzyme immunoassay (EIA) kit as per manufacturer’s instructions. The ELISA plates were read using Biotek Microplate Instrumentation (Winooski, VT). After spawning, eggs were collected and counted as per standard fish hatchery practice [25].

### Collection, Preparation of Tissue Samples and Histology

Fish were anaesthetized with clove oil (50 µl/l)) until they remained motionless. When there was cessation of heartbeat/respiration and the fish were unresponsive to external stimuli and had lost all reflexes, fish were removed from water. Ovary was dissected out and fixed in aqueous Bouin’s fluid for 24 hours. After fixing, tissues were washed in 70% alcohol in various changes till the yellow colour of picric acid was removed. Further, the ovaries were dehydrated in ascending grades of ethanol, cleaned in xylene and embedded in paraffin wax (58–60°C congealing point). From the paraffin block, section of 6–8 µm thickness were prepared using a rotary microtome and stretched on albumenised slide. The slides were fixed at 60°C overnight. The next morning, sections were deparaffinised in three changes of xylene and dehydrated in descending grades of alcohol to distilled water. The slides were stained in haematoxylin for 20 minutes, differentiated in 1% of alcohol and blued in ammonia water. After washing, sections were stained with eosin (working) for 10 minutes. Dehydrated and cleaned sections were then mounted in DPX [26] and observed under microscope to record gonadal changes through microphotography.

### Statistical Analysis

Data on serum hormone levels was subjected to analysis of variance (ANOVA), followed by Duncan’s Multiple Range Test with the help of SPSS-16.0 version software. All the data analysis was expressed as mean ± standard error.

## Results

### Functional Structures and Physicochemical Characterization of Nanoparticles

**Figure 1:**
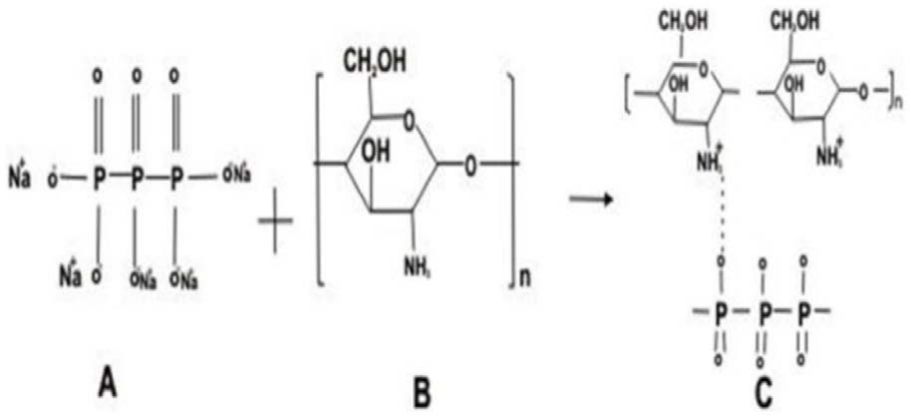
Schematic diagram of the functional structure of chitosan nanoparticles formed during ionic gelation process. (A) Sodium tripolyphosphate, (B) chitosan, (C) chitosan nanoparticles.

**Figure 2:**
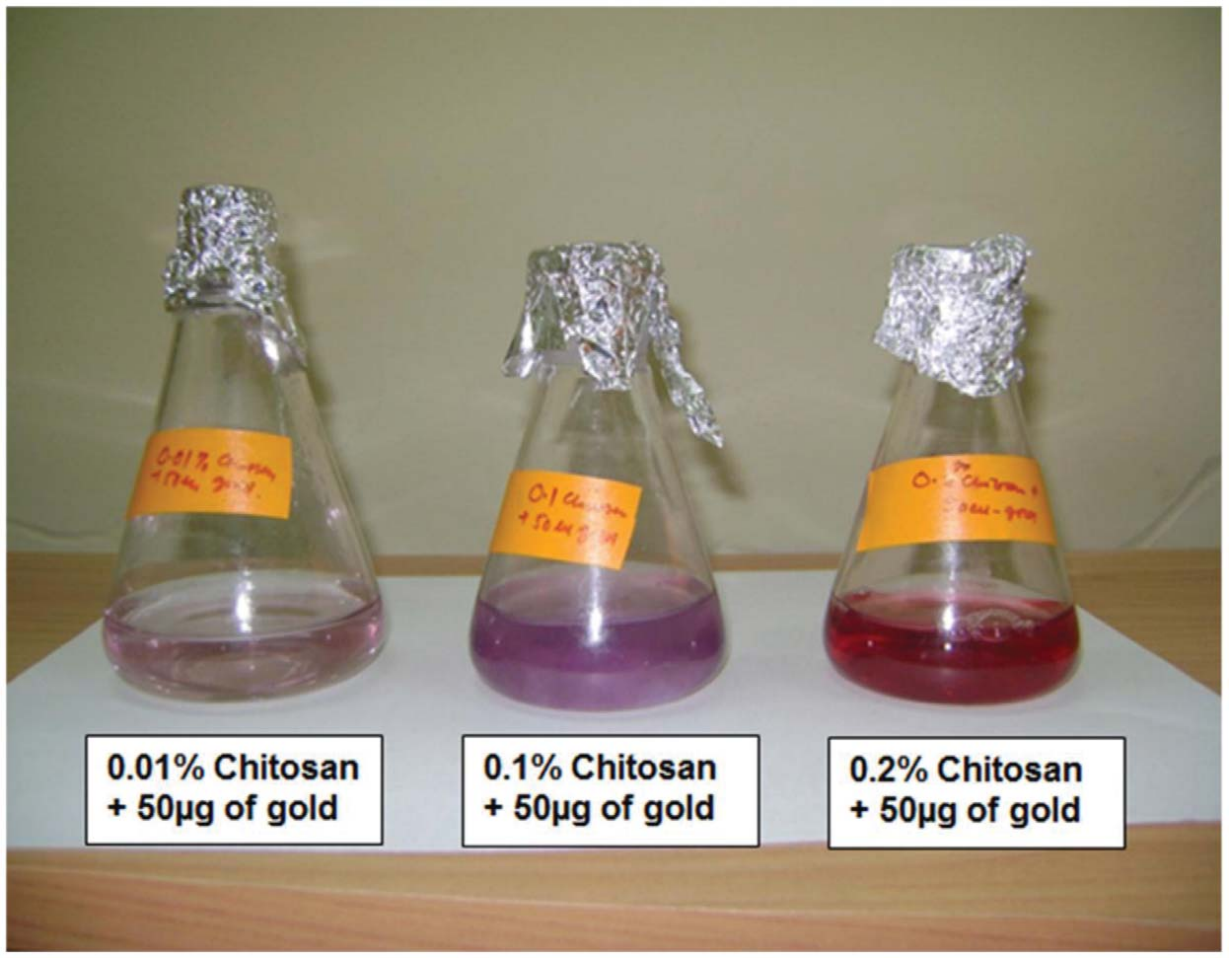
Formation of chitosan gold nanoparticles. Varying the ratio of gold salt to chitosan in the medium helps in achieving size controlled synthesis of nanoparticles. Nanoparticles formed in the last beaker were used for animal experimentation.

**Figure 3:**
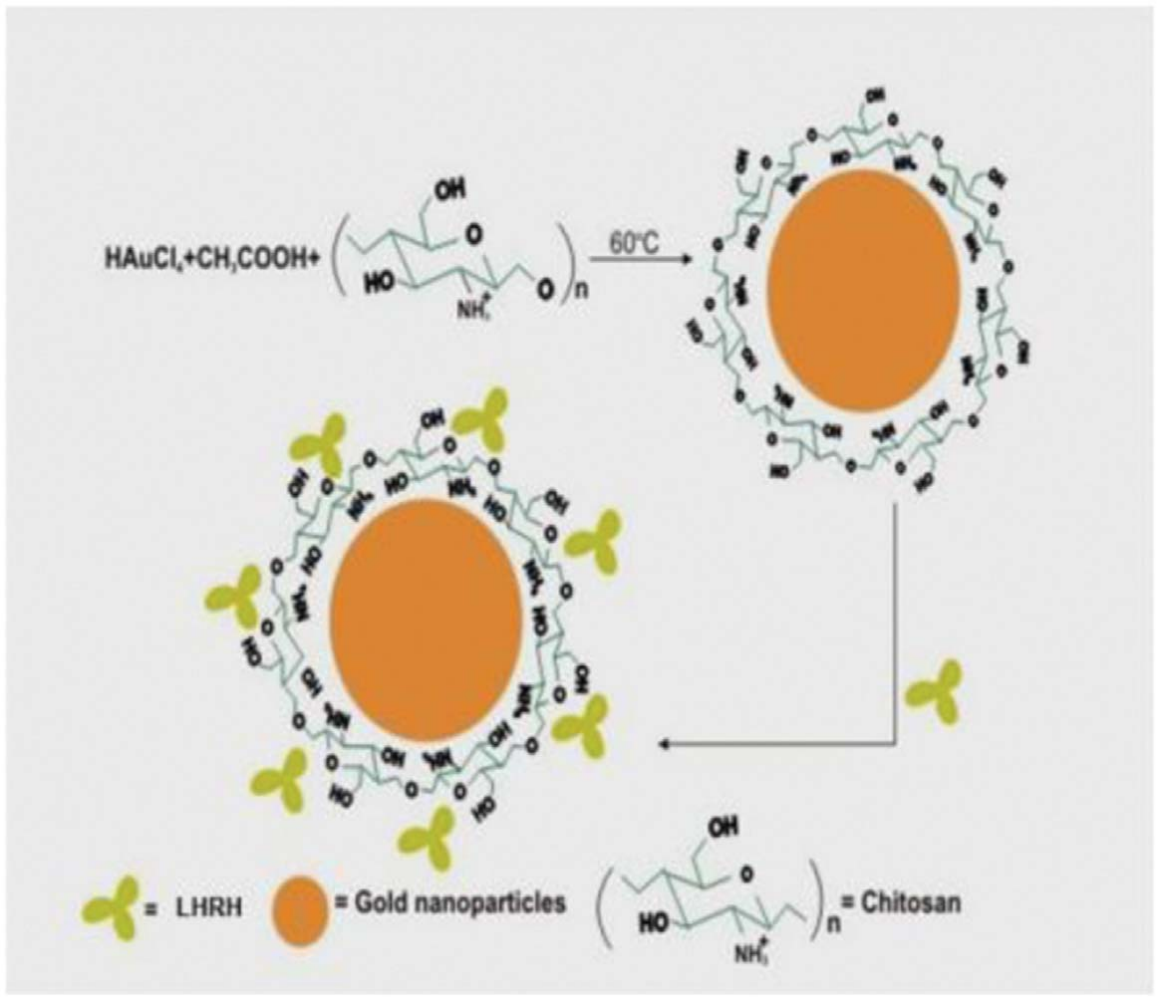
Schematic diagram showing the functional structure of chitosan encapsulated gold nanoparticles and subsequent conjugation of LHRH on chitosan encapsulated gold nanoparticles.

**Figure 4:**
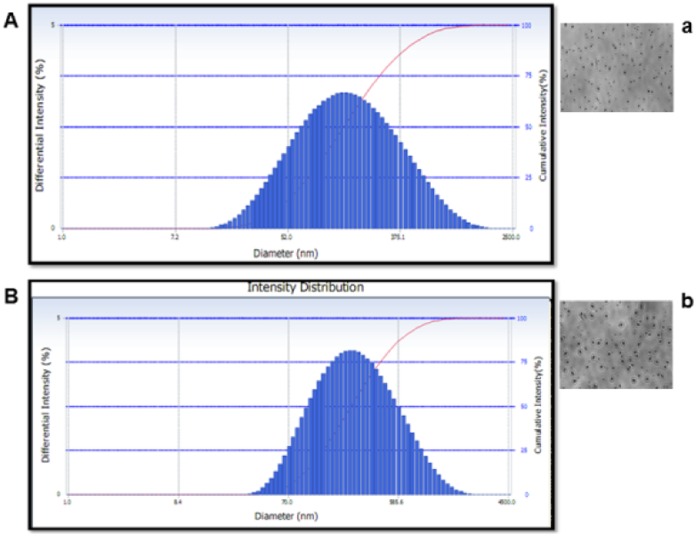
Particle size distributions of chitosan-LHRH (A) and chitosan-gold-LHRH nanoparticles (B) and their respective TEM Images (a, b).

Due to the reaction involving complex formation between oppositely charged species (negatively charged groups of the pentasodium tri-polyphosphate and the positively charged amino groups of chitosan), chitosan undergoes ionic gelation and precipitates to form spherical particles. The functional structure of chitosan nanoparticles is depicted in Fig. 1. In case of chitosan-gold nanoparticles, according to Turkevich, varying the ratio of gold salt to chitosan concentration in the medium helps in achieving size-controlled synthesis of nanoparticles and thus the formation of intense ruby red colour in beaker containing 0.2 per cent chitosan and 50 µg gold was indication of nano-size particles (Fig. 2), which were used for the animal experimentation. Thus, the solution containing 0.2% chitosan and 50 µg of gold was used for synthesizing LHRH preparation that was used for animal studies. Schematic diagram showing the functional structure of chitosan-gold-LHRH nanoparticles is shown in Fig. 3. It depicts chitosan encapsulation of gold nanoparticles and subsequent conjugation of LHRH on chitosan encapsulated gold nanoparticles. The distribution of particle size for chitosan-LHRH and chitosan-gold-LHRH nanoparticles is given in Fig. 4a and b. Chitosan-gold-LHRH nanoparticles were bigger (P<0.05) in size than chitosan-LHRH nanoparticles (Table 1). In general, both the particles were compact, spherical in structure, well dispersed and pretty stable as evinced by TEM and physico-chemical characteristics like polydispersity index (0.335 to 0.45) and zeta potential (−33.14 to −34.95 mV) (Table 1). Chitosan nanoparticles had 69 per cent, whereas chitosan-gold nanoparticles had 60 percent, entrapment efficiency for LHRH.

**Table 1:** Morphological and physico-chemical characteristics of salmon luteinizing-releasing hormone (LHRH) nanoparticles at 25°C in diluent water.

LHRH nanoparticles	Chitosan-conjugated	Chitosan- gold-conjugated
Size (nm)	114±10.3	192.5*±19.1
Zeta potential† (mV)	−33.14±6.67	−34.95±7.5
Polydispersity Index†	0.335	0.47
Entrapment efficiency (%)	69.00	60.00

† Size and zeta potential are expressed as mean± SE of n = 3.

* P<0.05 (by student t test).

### Serum Hormone Levels

**Figure 5:**
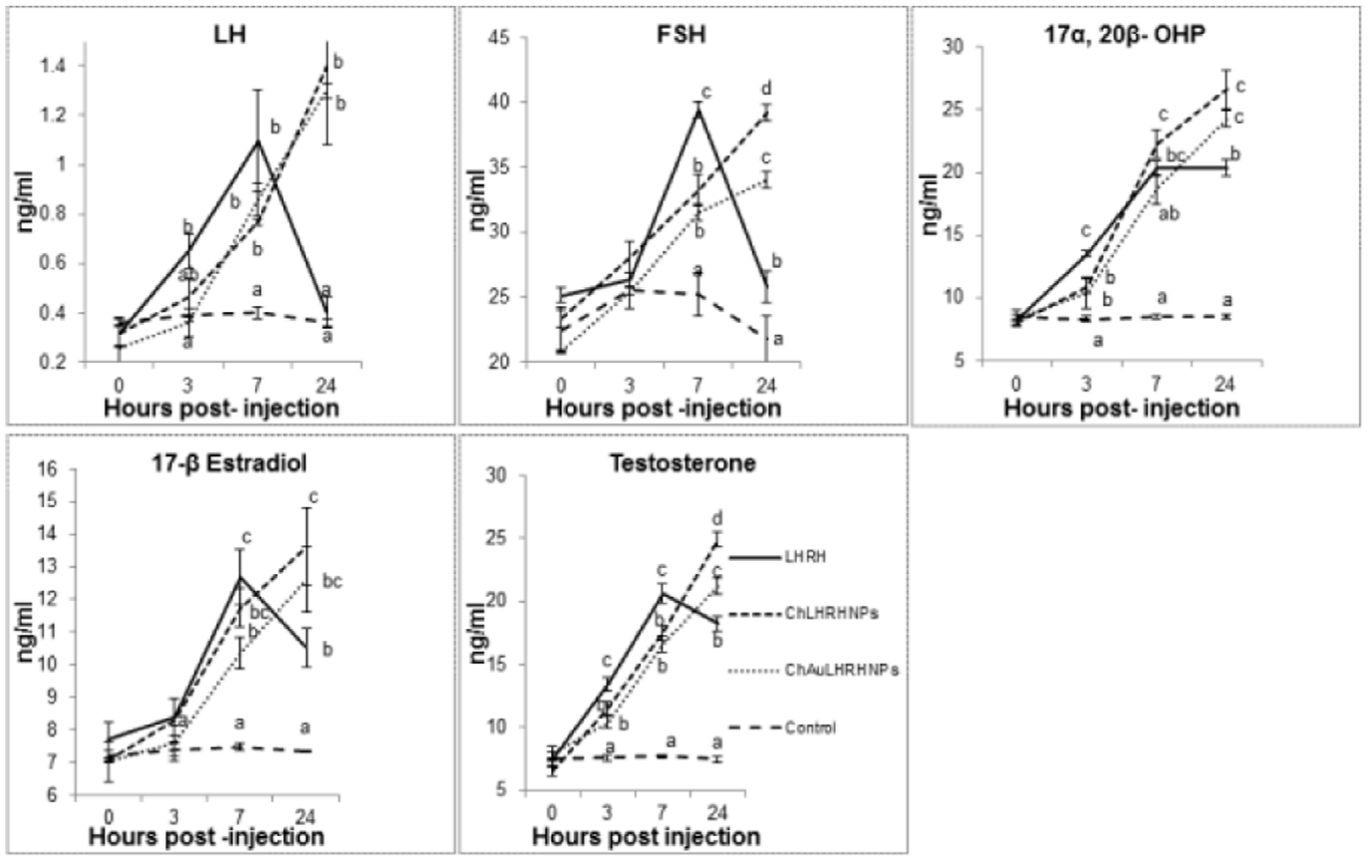
Serum levels of various reproductive hormones in female *Cyprinus carpio* following injection of different preparations of salmon luteinising hormone-releasing hormone (LHRH) during 24 hr period. Fishes were injected with either no active compounds (control) or with bare LHRH, chitosan LHRH nanoparticles (ChLHRHNPs) and chitosan-gold LHRH (ChAuLHRHNPs). Means bearing different superscript letters (a, b, c, d) differ significantly (P<0.01) at that hour point (vertical comparison). Each point is mean ±SE of three observations.

Significant effects of treatment (P<0.01) using nanoconjuagated hormone were discernible where increased levels of the hormone were recorded. Both the nanoconjugated preparations of LHRH induced sustained and controlled release of most of the hormones with highest (P<0.01) surge at 24 hrs. In case of bare hormone, surge due to LHRH reached its peak at 7 hrs and either remained at plateau for 17α, 20β-dihydroxy-4- pregnen-3-one (17α, 20β-OHP) and testosterone, or sharply declined for LH, FSH and 17β-estradiol (E2) (Fig. 5).

### Fertilization Rate

**Figure 6:**
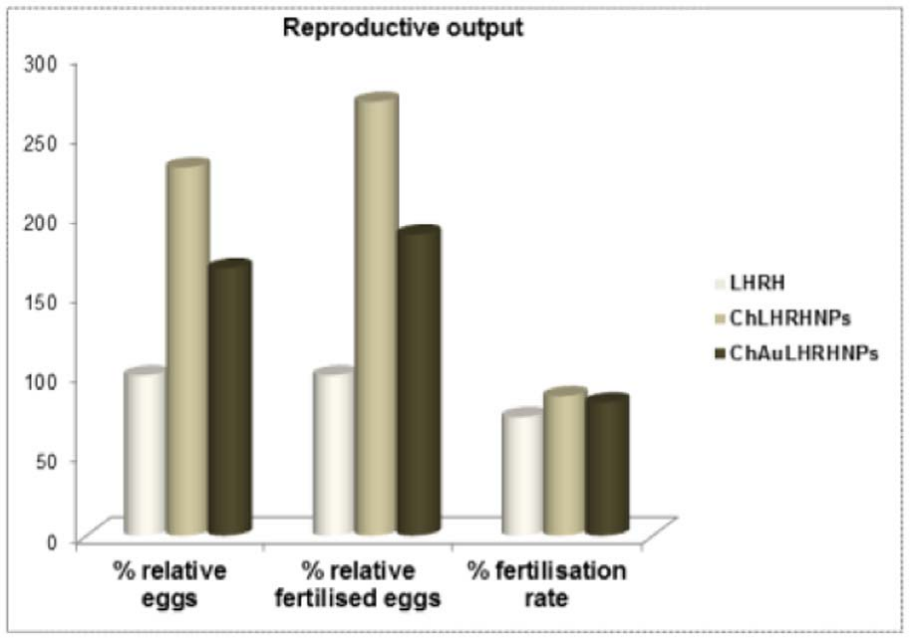
Reproductive output of the *Cyprinus carpio* injected with different preparations of salmon luteinising hormone-releasing hormone (LHRH). Percent relative eggs and fertilised eggs are expressed as relative to 100% in bare LHRH preparation. Fishes were injected with bare LHRH, chitosan LHRH nanoparticles (ChLHRHNPS) and chitosan-gold LHRH (ChAuLHRHNPs).

As fishes were reared in water appropriate for carp breeding (dissolved O_2_∶4 to 6 mg/L, temperature: 23 to 27°C, pH-7.2–8.3, total hardness: 243–254 mg/L, ammonia: 0.11–0.23, nitrite: 0.002–0.003 and nitrate: 0.02–0.04), and the hormonal preparations were effective, the spawning and fertilisation response was good. Out of total 348, 253.3 and 151.2 thousands eggs released following injections of bare LHRH, chitosan-nanoconjugated and chitosan-gold nanoconjugated LHRH 74%, 87% and 83% eggs were fertilised, respectively (Fig. 6). Thus, the pronounced effect of chitosan LHRH nanoparticles was evinced in terms of reproductive output.

### Histological Study of Gonads

**Figure 7:**
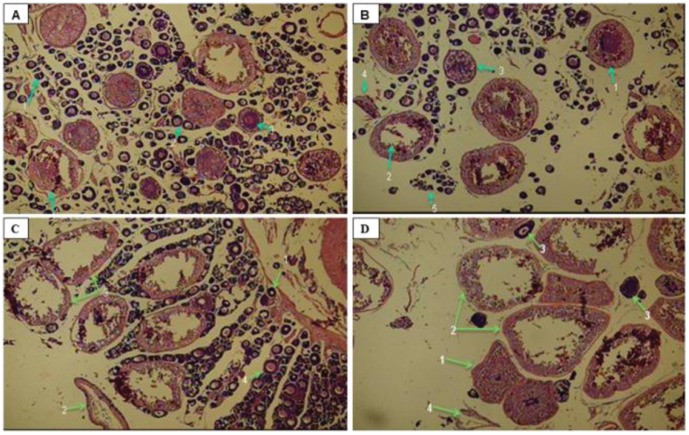
Histology of ovary from *Cyprinus carpio* injected with different preparations of salmon luteinising hormone- releasing hormone (LHRH). A]. from control fishes: 1.Ovigerous lamellae with immature oocyte; 2. mature oocyte; 3. maturing oocyte; 4. atretic oocyte. B] from fish injected with bare LHRH. 1. mature/ripe oocyte; 2. Partially spent oocyte; 3. cortical alveoli stage oocyte; 4. spent follicle; 5. immature oocytes in nest C] from fish injected with chitosan LHRH nanoparticles. 1. Oocytes nested in lamellae 2. spent follicle 3. spent oocytes 4. ovigerous lamellae with immature oocytes D] from fish injected with chitosan gold LHRH nanoparticles 1. mature oocytes 2. spent oocytes 3. immature oocytes 4. discharged follicle.

The histology photographs of ovaries from different groups are given in Fig. 7. Histological studies of gonads in control fish showed no spawning and the matured ova were seen with yolk globules. The fish injected with LHRH showed partial spawning with many fully matured ova. However, complete spawning occurred as evidenced by the presence of spent ova and empty follicles in the ovaries of both the chitosan-LHRH and chitosan-gold LHRH nanoparticle-injected fish.

## Discussion

The present work was carried out to develop the nanodelivery of the hormone in fish for obtaining a long lasting surge of GnRH/LHRH. This nanoconjugation increases the stability of the hormone, which otherwise has short lifetime in the blood [2]. Such nanodelivery has quite a pronounced advantage over multiple injections. Various chitosan-conjugated nanoparticles of LHRH have been developed for treating the diseases such as steroid hormone disorders [27] for terrestrial species. However, this is the first report on development of chitosan-nanoconjugated hormone for its application in fish reproduction. Two types of salmon LHRH nanoparticles were developed using chitosan and chitosan-gold as a nanocarrier. The ionotropic gelation method used in the present study has already been successfully employed to prepare chitosan nanoparticles for the delivery of peptides and proteins [28].

During synthesis, intense ruby red colour formation in case of chitosan-gold nanoparticles was a clear indication of development of nanoparticles. A similar observation was also made by Turkevich [20]. In the present work, chitosan-gold nanoparticles were seen changing colour from whitish to purple and then ruby red colour. In a study by Bhumkar et al. [29] chitosan concentrations of 0.1% and above employed for reduction and synthesis of gold nanoparticles showed no aggregation, while lower concentrations of 0.01% to 0.05% resulted in aggregated particles. Hence, they used 0.2% chitosan reduced gold nanoparticles for loading of insulin because of the best zeta potential, stability (6 months) and viscosity of the various gold nanoparticles prepared from these, which showed effective glucose control in diabetes in rats. In another study [30], out of three different chitosan concentrations (0.1, 0.2 and 0.3 per cent), optimum encapsulation and overall release of protein was found with nanoparticles prepared from 0.2% chitosan. In yet another study, hepatocyte growth factor incorporated (0.2%) chitosan nanoparticles were found to augment the differentiation of stem cells into hepatocytes for the recovery of liver cirrhosis in mice [31]. Thus, our selection of 0.2% chitosan nanoparticles corroborate with published reports [29]–[31]. The chitosan and chitosan-gold preparations of LHRH were nanosize as evidenced by the particle size analyzer and TEM image. The larger (P<0.05) size of chitosan-gold nanoparticles with 9% less entrapment efficiency than chitosan nanoparticles was obvious because of the usage of gold in addition to chitosan. The mean size of estradiol-loaded chitosan nanoparticles prepared [18] using the same technique was 269 nm with a zeta potential of +25.4 mV and entrapment efficiency of 67%. The nanoparticles developed in our study were much smaller in size and more stable than these reported [18]; [30]. Our result is comparable with reported [21] particle size of 115 nm with 51% entrapment for estradiol loaded PLGA nanoparticles. While polydispersity index (PDI) of chitosan-gold-LHRH nanoconjugates was slightly higher (0.47) than chitosan-LHRH nanoconjugates (0.33), the PDI and functionality was well in accordance with published reports [30], [32]. Out of twenty nanoformulations prepared with different concentrations of plasmid DNA and chitosan for expression of interleukin-12 (IL-12) having antitumor effect, the chitosan-DNA nanoparticles that had minimal cytotoxicity and suggested as suitable candidate for IL-12 gene delivery had mean particle size of 381.83±82.77 nm, PDI of 0.44±0.066 and encapsulation efficiency of 82.17±5.61 [32]. In studies by Dounighi et al. [30], out of three different nanoproteins, the best (0.2%) chitosan-loaded protein nanoparticles (in terms of encapsulation and protein release) were 370±34.7 nm in size and had PDI of 0.429, while PDI of nanoparticles with 0.1% chitosan concentration was not even within the acceptable range, as it formed aggregates with large diameters.

The present hormonal and reproductive output studies suggest that the common carp pituitary is receptive to bare and nanoconjugated LHRH, which is evidenced by hormone levels in treated fish. Spawning experiments under routine hatchery conditions with a single hormonal injection [23] resulted in an initial rise in the level of maturational gonadotropin (GtH) 3 hrs after treatment reaching peak at 14 hrs, when full ovulation took place, as reflected by the presence of expelled eggs on the bottom of the tank. This rise was associated with increased levels of 17β-estradiol (E_2_) and 17α, 20β-OHP. Physiological increases in serum level of gonadotropins (FSH, LH), estadiol and 17α, 20β-OHP in *C. carpio* in the current study is in agreement with that of Drori et al. [23]. Moreover, the effect of the size of the nanoparticles on endogenous hormone levels (LH, FSH, 17α, 20β-OHP, estradiol and testosterone) and duration of release has already been reported [17]. The observed response to nanoconjugated preparations of LHRH in terms of the levels of these hormones is in agreement with the previous studies. Compared to the bare LHRH-administered group where hormonal peaks was found to decrease after 7 hrs post injection, sustained and controlled release of the endogenous hormone showing enhanced peak at 24 hrs were indicative of resistance of chitosan- and chitosan-gold nanoparticles against enzymatic degradation. High drug loading and prolonged drug release are the advantages of using chitosan for delivery of biomolecules [33]. Chitosan nanoparticles are promising carriers for protein delivery [34] because of the solubility of chitosan in an aquatic medium resulting in better permeability. A similar permeation enhancing effect of chitosan on human insulin has been reported [35]–[36]. It was expected that the efficacy of nanoform of LHRH would surpass the bare LHRH. Indeed, despite injecting half the dose of bare LHRH in nanoconjugated forms (either with chitosan or chitosan-gold), elevated and sustained surge of steroid hormones, especially gonadotropins (FSH and LH), were observed. This resulted in an increase in follicle maturation and subsequent ovulation, leading to high reproductive output in the form of total and fertilized eggs. The nano-form of LHRH could penetrate deep into tissues through fine capillaries, cross the epithelial lining, and is taken up efficiently by the cells [37]. Elevated testosterone and estradiol levels during vitellogenic growth and ovulation found in this study are consistent with published reports in many species [38]–[40]. Plasma 17α, 20β- OHP levels in nano LHRH-injected fish followed the same trend as estradiol and testosterone. This is consistent with many studies with teleosts that suggest the involvement of 17α, 20β-OHP in final oocyte maturation [41]. Our results on sustained elevated levels of LH over a period of 24 hrs corroborate well with a published report [42], in which enhanced reproductive output (milt production) in spermiating males of European sea bass (*Dicentrarchus labrax*) for six weeks with controlled-release of GnRH analogue was found. Compared to bare LHRH preparation, the per cent relative eggs (total and fertilised) produced by fish injected with chitosan LHRH nanoconjugates (130 and 171 per cent) and chitosan-gold nanoconjugates were more (67/88 per cent). Sustained delivery systems of gonadotropin-releasing hormone analogue [43] have been successfully used to synchronize females for reproduction with better quality egg production, a 1.75 times increase in fertilization and a 2.4 times greater hatching rate in female yellowtail flounder (*Pleuronectes ferrugineus*).

No adverse effect of gold nanoparticles was discernible through histological examination. In spite of repeated administration of higher dose in mice, no toxicity of gold nanoparticles was found in mice [44]. Bhumkar et al. [29] reported improved pharmacodynamics with transdermal application of chitosan reduced gold nanoparticles loaded with insulin in controlling the postprandial hyperglycemia. And unlike in diabetes, which calls for repeated administration of chitosan-gold insulin nanoparticles, there is greater safety with a single injection of chitosan-gold hormonal nanoparticles. Moreover, these can be used in diseases for which only a few injections are required to treat.

### Conclusions

The chitosan- and chitosan-gold nanoconjugates of salmon leutinizing hormone releasing hormone increased the surge of gonadotropin levels in *Cyprinus carpio*. The sustained release of these nanoconjugated hormones resulted in good reproductive output with no toxic effect. This establishes the potential of chitosan- and chitosan-gold conjugated hormone nanoparticles as a new means of manipulating reproduction in fish. The low cost chitosan-conjugated LHRH may be useful for overcoming reproductive problems in fishes. Such nanodelivery has quite a pronounced advantage over multiple injections. This is the first comprehensive report on development of chitosan- and chitosan-gold nanoconjugated hormone for its application in animal reproduction.
